# Learning curve for robotic rectal cancer resection at a community-based teaching institution

**DOI:** 10.1007/s11701-023-01671-2

**Published:** 2023-11-03

**Authors:** Kristen Coleman, Angela N. Fellner, Hamza Guend

**Affiliations:** 1 Department of Surgery, TriHealth, 375 Dixmyth Ave, Cincinnati, OH 45220 USA; 2 TriHealth Hatton Research Institute, Cincinnati, OH USA; 3 TriHealth Surgical Institute, Cincinnati, OH USA

**Keywords:** Rectal cancer, Learning curve, Robotic surgery

## Abstract

The surgical management of rectal cancer is shifting toward more widespread use of robotics across a spectrum of medical centers. There is evidence that the oncologic outcomes are equivalent to laparoscopic resections, and the post-operative outcomes may be improved. This study aims to evaluate the learning curve of robotic rectal cancer resections at a community-based teaching institution and evaluate clinical and oncologic outcomes. A retrospective review of consecutive robotic rectal cancer resections by a single surgeon was performed for a five-year period. The cumulative sum (CUSUM) for total operative time was calculated and plotted to establish a learning curve. The oncologic and post-operative outcomes for each phase were analyzed and compared. The CUSUM learning curve yielded two phases, the learning phase (cases 1–79) and the proficiency phase (cases 80–130). The median operative time was significantly lower in the proficiency phase. The type of neoadjuvant therapy used between the two groups was statistically different, with chemoradiation being the primary regimen in the learning phase and total neoadjuvant therapy being more common in the proficiency phase. Otherwise, oncologic and overall post-operative outcomes were not significantly different between the groups. Robotic rectal resections can be done in a community-based hospital system by trained surgeons with outcomes that are favorable and similar to larger institutions.

## Introduction

Robotic surgery continues to gain popularity for various oncologic surgical resections. This trend is notable in colorectal surgery and more specifically, rectal cancer resections. Advantages of robotic technology include improved dexterity, stable retraction, three-dimensional visualization, ability to work in multiple quadrants, improved ergonomics, and flexibility in stapling devices. Resection for rectal cancer presents additional challenges specific to the importance of preserving oncologic quality, such as circumferential resection margin, completeness of total mesorectal excision (TME), and distal margin. Use of the robotic platform has been shown to have comparable oncologic outcomes to laparoscopy, including lymph node harvest and circumferential and distal resection margin positivity [1–3]. It has also been shown to have decreased rate of conversion to open [1–3], decreased length of stay (LOS) [3], and less time to return of bowel function [3]. There has been no significant difference in mortality or leak rate between laparoscopic and robotic approaches [1, 3].

With increased utilization of the robotic platform, several studies have been published evaluating learning curves for colorectal surgeons [4–13]. The learning curves are established using the cumulative sum (CUSUM) analysis described by Bokhari et al. [6]. Most of the studies were performed at large institutions, and the time intervals used to assess learning curve vary widely. The available papers report on both single and multiple surgeons’ learning curves, and there were generally no significant differences in post-operative and oncologic outcomes between the different phases of learning. Some of the published data suggests that there is a shorter learning curve for robotic rectal resection than laparoscopic and even open surgery [4, 8].

Data shows significantly lower use of minimally invasive approaches for rectal cancer resection at community and comprehensive community hospitals versus academic institutions [14, 15]. These studies also found higher rates of conversion to open, increased 90-day mortality and worse oncologic outcomes, such as margin positivity and increased incomplete lymph node harvest in minimally invasive rectal cancer resection at community/comprehensive community hospitals.

In this study we aim to evaluate the learning curve for robotic rectal cancer resections performed at a community teaching hospital based on retrospectively collected operative, pathologic, and outcomes data. The primary outcome being assessed is change in total operative time for consecutive robotic rectal resections. This will be displayed as a CUSUM analysis. The secondary outcomes are oncologic outcomes across learning phases, 30-day peri-operative variables and complications.

## Materials and methods

Institutional Review Board (IRB) approval was obtained from the TriHealth IRB (Cincinnati, OH) and need for informed consent was waived. This was a retrospective review of consecutive robotic rectal resections performed by a single surgeon over a five-year period from January 1, 2016 to December 31, 2021. All resections were performed for a diagnosed malignancy and were done electively. The operations took place at two hospitals within a single community-based teaching institution. All data was collected from patients’ electronic medical records, including operative and pathologic reports. Exclusion criteria were laparoscopic resections, age less than 18 years old, emergent cases, or cases performed for any disease other than rectal adenocarcinoma. All cases were performed robotically including splenic flexure mobilization when necessary, as well as utilization of all robotic instrumentation such as robotic staplers and vessel sealer. The three operation types included were low anterior resection (LAR), abdominoperineal resection (APR), and proctectomy with creation of end colostomy (also referred to as a low Hartmann’s).

Pre-operative variables included demographics such as age, gender, body mass index (BMI), American Society of Anesthesiologists (ASA) score, diagnosis, neoadjuvant therapy, tumor location. Neo-adjuvant therapy included chemo-radiotherapy, total neo-adjuvant therapy (TNT), or short course radiotherapy. Operative details included the operation performed, total operative time from the first incision until closure, ostomy creation (ileostomy or colostomy), anastomotic details (hand-sewn versus stapled), conversion to open, estimated blood loss (EBL), and any intra-operative complications. Pathologic variables included final pathologic staging, both circumferential resection margin (CRM) and distal margin status, lymph node harvest, lymphovascular invasion (LVI), and completeness of TME graded as complete, incomplete or near complete by a gastrointestinal trained pathologist. Post-operative complications included major complications, defined as anastomotic leak or deep abdominopelvic abscess; and minor complications, which included anemia requiring transfusion, surgical site infection (SSI), ileus requiring NG tube placement, acute kidney injury (AKI), urinary tract infection (UTI), urinary retention, cardiac complications, and pulmonary complications. Other post-operative outcomes included length of stay (LOS), 30-day mortality, readmissions, and return to the operating room.

### Statistical analysis

A CUSUM analysis was performed to evaluate operative times as the cases progressed chronologically. The operative times were calculated from skin incision to incision closure in minutes and plotted on a line graph to be used in the CUSUM analysis. The CUSUM is a running total of the differences between each consecutive data point and the overall mean operative time. The equation we used was described initially by Bokhari in 2011 and has been used in multiple learning curve papers since [6]. The CUSUM for the first case is equal to its operative time (OT) minus the overall mean operative time (MOT). The CUSUM for all subsequent cases followed the equation:

$${\text{CUSUM}}_{{\text{current case}}} = {\text{ CUSUM}}_{{\text{prior case}}} + \, \left( {{\text{OT}}_{{\text{current case}}} - {\text{MOT}}} \right).$$

These numbers were plotted on a line graph, thereby giving rise to the curve. The phases of the learning curve, learning and proficiency, were then determined based on a steep drop-off at case number 79.

Next, peri-, intra-, and post-operative variables were compared between patients in the learning and proficiency phases. Normal scale variables were analyzed using Student’s *t*-test; non-parametric scale variables were analyzed using the Mann–Whitney *U*-test. Categorical variables were analyzed using the Pearson chi-square or Fisher’s Exact test. Alpha was set at *p* ≤ 0.05 to determine statistical significance.

## Results

A total of 131 patients were evaluated for inclusion, all of whom underwent elective robotic rectal resection for a rectal cancer. One patient with a gastrointestinal stromal tumor was excluded. All 130 patients in the final analysis had a rectal adenocarcinoma that was resected electively using the DaVinci robotic platform (Intuitive Surgical Company, Sunnyvale, CA USA).

Pre-operative baseline characteristics and demographics were similar between groups, with the only exception being more ASA category IV patients in the learning phase (5 vs 0). However, this was not significantly different overall between the two groups (Table1). One patient in the learning phase and two in the proficiency phase had a diverting colostomy for near obstruction/obstruction prior to their elective resection. These colostomies were reversed during their definitive operation. One patient had an anastomotic recurrence during the study period and was re-operated on. The data from both operations is included.

**Table 1:** Demographics/baseline characteristics

Variable	Learning phase n (%)	Proficiency phase n (%)	*p*-value
Sex			
Male	53 (67.1)	33 (64.7)	0.462
Female	26 (32.9)	18 (35.3)	
Age, years, mean (SD) age	61.3 (10.4)	59.7 (10.8)	0.406
BMI, (kg/m^2^), median (IQR)	29 (25.6, 33.5)	28.6 (25.2, 32.3)	0.441
ASA	N (%)	N (%)	
II	31 (39.2)	24 (47.1)	0.158
III	43 (54.4)	27 (52.9)	
IV	5 (6.3)	0	

**Fig. 1:**
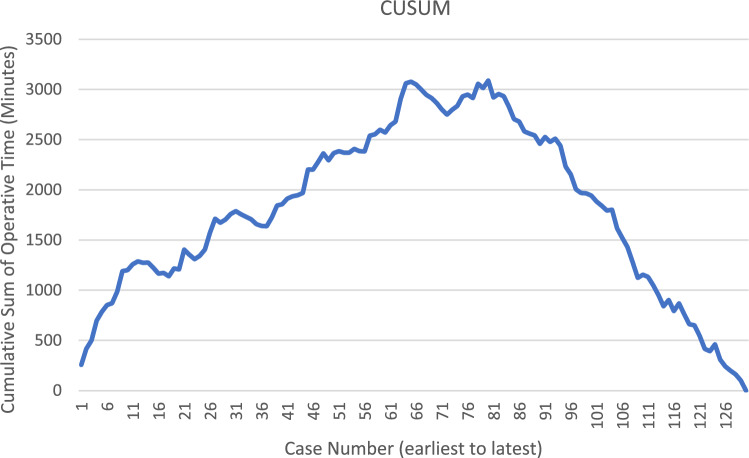
Learning curve based on cumulative summation (CUSUM) method

The primary outcome of this study was the learning curve of robotic rectal cancer resections based on total operative time. When plotted on a chart versus time, the operative times showed highest times early in the study period which gradually decreased over time. This was also supported by the statistical analysis, which showed that the median (interquartile range, IQR) operative time was significantly lower in the proficiency phase [340 min (296, 400) vs. 260 min (216, 300)] (*p* < 0.0001) (Table 1). A CUSUM analysis was performed using the above equation and the numbers were plotted on a line graph to establish the learning curve (Fig. 1). The sharp drop of the curve at case 79 is considered the cutoff between learning and proficiency phases.

Next, we compared outcomes between the learning and proficiency phases. Significantly more patients in the proficiency group had neoadjuvant treatment of any kind (*p* < 0.0001) (Table 2). However, when compared by type of neoadjuvant treatment, statistically more patients in the learning phase received neoadjuvant chemoradiation (*p* < 0.0001), while more patients in the proficiency phase received TNT (*p* < 0.0001). No significant difference was reported between the groups for other neoadjuvant treatments, such as single therapy chemo or radiation. There were more distal tumors in the learning phase (20 vs 8), but there was no significant difference in the distribution of tumor location between the groups (*p* = 0.222).

**Table 2:** Pre-operative variables

Pre-operative variable	Learning phase n (%)	Proficiency phase n (%)	*p* value
Neoadjuvant therapy	63 (79.7)	45 (88.0)	** < 0.0001**
Chemoradiation	52 (65.8)	17 (33.3)	** < 0.0001**
Total neoadjuvant therapy (TNT)	7 (8.9)	24 (47.1)	** < 0.0001**
Other neoadjuvant^a^	5 (6.3)	4 (7.8)	0.499*
No neoadjuvant therapy	15 (19.0)	6 (11.8)	
Tumor location^b^			
Upper	30 (38.0)	17 (33.3)	0.222
Mid	29 (36.7)	26 (51.0)	
Distal	20 (25.3)	8 (15.7)	

^a^Other neoadjuvant includes short course radiation alone or chemo only in patients who had a history of prior pelvic irradiation

^b^Tumor location determined based on staging rectal cancer specific pelvic MRI

Fewer APRs were performed in the proficiency phase (9 vs 4), but the type of operation performed between groups was not significantly different (Table 3). The use of diverting loop ileostomy (DLI) and conversion to open were similar between the groups. There were more combined cases in the learning phase (11.4% vs 5.9%) but this was not significantly different (*p* = 0.230). Only two intra-operative complications were reported, one being a ureteral injury in the learning phase and a case of acute limb ischemia in the proficiency phase.

**Table 3:** Operative variables and intraoperative complications

Operative variables	Learning phase n (%)	Proficiency phase n (%)	*p* value
Procedure			
Low anterior resection (LAR)	65 (82.3)	44 (86.3)	0.795
Abdominoperineal resection (APR)	9 (11.4)	4 (7.8)	
Low Hartmann’s	5 (6.3)	3 (5.9)	
Anastomotic type (if applicable)	65	43	0.452
Hand-sewn	3 (4.6)	3 (7.0)	
Stapled	62 (95.4)	40 (93.0)	
Stoma	67	46	0.502
Ileostomy (% within phase)	53 (67.1)	39 (76.5)	
Colostomy	14 (17.7)	7 (13.7)	
Conversion to open	5 (6.3)	2 (3.9)	0.434
Operative time (min), median (IQR)	340 (296, 400)	260 (216, 300)	< 0.0001
Estimated blood loss (ml), median (IQR)	150 (100, 300)	125 (75, 250)	0.391
Intra-operative complications			
Ureteral injury	1 (1.2)	0 (0)	
Acute limb ischemia	0 (0)	1 (2.0)	
Combined case^a^	9 (11.4)	3 (5.9)	0.230

^a^Combined cases include those which required participation of a surgeon from a different specialty, including gynecology, urology, and hepatobiliary

Oncologic outcomes are presented in Table 4. In both groups, the majority of tumors were T3 on final pathology T-staging (41.8 and 47.0%), and there was no significant difference in the overall pathologic staging between groups (*p* = 0.552). Additionally, no significant difference was observed in the CRM positivity, distal margin positivity or TME completeness between the groups. However, in the learning phase there were multiple pathology reports that did not document the CRM or TME. The presence of lymphovascular invasion was significantly more common in the learning phase (13.9 vs 2.0%, *p* = 0.049). The median (IQR) number of lymph nodes harvested was also similar between the groups.

**Table 4:** Oncologic outcomes

Oncologic outcomes	Learning phase, n (%)	Proficiency phase, n (%)	*p* value
Pathologic stage			0.552
Depth of invasion (T-stage)			
T0 (complete pathologic response)	21 (26.6)	8 (15.7)	
Tis/1	7 (8.9)	3 (5.9)	
T2	14 (17.7)	14 (27.4)	
T3	33 (41.8)	24 (47.0)	
T4	4 (5.1)	2 (3.9)	
Nodal metastasis			
N0	50 (63.3)	39 (76.5)	
N1	22 (27.8)	11 (21.6)	
N2	7 (8.9)	1 (2.0)	
Distant metastasis			
M0	74 (93.7)	50 (98.0)	
M1	5 (6.3)	1 (2.0)	
Margin positivity			
Circumferential resection margin (CRM)	4 (5.3)	0 (0)	0.124
Distal	1 (1.3)	0 (0)	0.608
Lymphovascular invasion	10 (13.9)	1 (2.0)	0.049
TME completeness^a^			
Complete	47 (79.7)	47 (92.2)	0.170
Near complete	10 (16.9)	3 (5.9)	
Incomplete	2 (3.4)	1 (2.0)	
Not reported	20 (25.3)	0 (0)	
Lymph node harvest, median (IQR)	18 (13, 26)	19 (14, 26)	0.704

^a^Percentage refers to the number of pathology reports that commented on TME, not the total number of patients

There were no significant differences in any of the 30-day post-operative adverse outcomes, LOS, re-admission, re-operation, or mortality (Table 5). Acute kidney injury approached significance, however, with a rate of 13.9% in the learning phase vs 3.9% in the proficiency phase (*p* = 0.055). The ileus rate was 21.5% in the learning phase but was not significantly different from the proficiency phase at 11.8% (*p* = 0.116).

**Table 5:** 30-day post-operative complications and outcomes

Post-operative outcome	Learning phase n (%)	Proficiency phase n (%)	*p* value
Major complication			
Anastomotic leak/deep abscess	4 (5.1)	4 (7.8)	0.386
Minor complication			
Anemia requiring blood transfusion	6 (7.6)	2 (3.9)	0.325
Surgical site infection (SSI)	1 (1.3)	0 (0)	0.608
Ileus (requiring NG)	17 (21.5)	6 (11.8)	0.116
Acute kidney injury	11 (13.9)	2 (3.9)	0.055
Urinary tract infection	7 (8.9)	2 (3.9)	0.238
Urinary retention	6 (7.6)	3 (5.9)	0.501
Cardiac complication	5 (6.3)	1 (2.0)	0.239
Pulmonary complication	1 (1.3)	0 (0)	0.608
Other complication^a^	4 (5.1)	1 (2.0)	0.346
Median (IQR) LOS (days)	4 (3, 6)	4 (3, 5)	0.522
30-day mortality	0 (0)	0 (0)	n/a
30-day return to OR	5 (6.3)	7 (13.7)	0.134
30-day re-admission	13 (16.5)	7 (13.7)	0.437

^a^Other complications include: seizure, SMV thrombosis, missed enterotomy, lower extremity compartment syndrome in learning phase and ureteral injury in proficiency phase

## Discussion

The total operative time/learning curve analysis in this study demonstrates improvement in time over the study period (Fig. 1). The median operative time in the learning phase of 340 min was higher than the 298.5 min reported in the ROLARR trial [18] and 339.2 min reported by Kim et al. [17]. The median time in our proficiency phase was much lower than both at 260 min. In addition, we included co-cases/multi-visceral resection in our data, which can often significantly impact operative time.

A CUSUM analysis was then performed to demonstrate the learning curve. Most CUSUM analyses included three learning phases, including a plateau phase between learning and expert/proficiency phases. There was a short plateau period from case 64–79, after which the curve drops quickly. Given the small number of cases in this potential plateau phase, these cases were considered part of the learning phase rather than a separate phase. We consider the sharp drop after case 79 as the cutoff between the learning and proficiency phases. The number of cases required to get to proficiency/expert level in the available literature ranges widely, from 10’s to > 100 for some surgeons [4–13].

Many factors can contribute to the overall operative time including tumor size, location, T-stage, multi-organ resection, experience of the operating room team, and resident involvement. We elected to utilize total operative time as it truly reflects all components of the overall resection such as specimen extraction, anastomotic technique, and endoscopic evaluation of the anastomosis, but also includes the docking, which is a crucial part of the learning experience for robotic surgery. Additionally, most of our cases involved a resident on a teaching console which certainly can influence the operative time.

Patients in the two groups received a variety of neoadjuvant therapy regimens. In total, 79.7% (learning) and 88.0% (proficiency) received some form of neoadjuvant therapy, with a *p* < 0.0001. 65.8% of those in the learning phase received chemoradiation, compared to 33.3% in the proficiency group (*p* < 0.05). Total neoadjuvant therapy (TNT), or chemotherapy plus chemoradiation, became the standard treatment over time, and 47.1% of patients in the proficiency group had this therapy vs only 8.9% in the learning phase (*p* < 0.0001). The standard neoadjuvant therapy drastically changed somewhere around the end of the learning phase and beginning of the proficiency phase. The notable difference reflects the ever-evolving management of cancer in general, but especially the rapid advances in management of rectal cancer.

As far as type of operation performed, there was no significant difference between the two groups. APRs were performed in patients in whom a ~ 1 cm distal margin could not be obtained and low Hartmann’s procedures were performed selectively in patients with low tumors and poor sphincter function who we believed would have better functional outcomes with an end colostomy rather than an ultralow anastomosis. There was no difference in the rate of stoma creation between the two groups, the majority in both being loop ileostomies. The colostomies were all end colostomies that were created in patients who underwent APR or low Hartmann’s, with the intention that they would all be permanent. DLI rate in the learning group was comparable to the ROLARR trial, in which 60.2% of patients had a DLI created. The conversion to open rate was 6.3% and 3.9% in the learning and proficiency phases, respectively (*p* = 0.434). The conversion rate in the learning group was on par with and the proficiency group improved compared with those reported by two meta-analyses evaluating the outcomes with robotic rectal resections, which were 5.72% [2] and 7.3% [1].

Oncologic outcomes (Table 4) were not significantly different between the two phases. Three patients in the learning phase did not have CRM reported on pathology. Lymphovascular invasion was significantly higher in the learning group on final pathology (*p* = 0.049); however, 7 of these pathology reports did not comment on lymphovascular invasion. The clinical significance of this difference is uncertain. The difference in TME quality was difficult to assess due to nearly 25% of pathology reports in the learning phase not reporting on this. The pathology reports have since become standardized throughout the institution, and all pathologic data was complete by the proficiency phase. We therefore calculated the rates of complete, near complete and incomplete TME by accordingly using the total number of reports that included TME in the learning group as the denominator. With this, there was an increase in the rate of complete TME to 92.2% vs 79.7% and a decrease in the rate of near complete (5.9% vs 16.9%) and incomplete TME (2.0% vs 3.4%) in the proficiency phase vs learning. Though it cannot be truly assessed for statistical significance, we believe this is clinically significant and is likely attributable to better operative technique as we progressed through the learning curve. Additionally, given the favorable clinical outcome associated with complete and near complete TME, we do not believe oncologic outcomes are compromised during the learning phase. When comparing our outcomes with those of large RCTs analyzing robotic rectal resections, they are all comparable or improved. The rate of complete TME in this analysis was much higher in the proficiency group (92.2%) and comparable in the learning group (79.2%) to the ROLARR (75.4%) and Kim studies (80.3%).

Complete pathologic response, or T0N0 on final pathology, was recorded in 26.6% and 15.7% of the patients in the learning and proficiency groups respectively. The “watch and wait” approach to patients with clinical complete response to neoadjuvant therapy has continued to have promising results for organ preservation [18]. We have adopted a similar approach for highly selected patients which could potentially explain this decrease in pathologic complete response rate. In our patient population, lymph node harvest was very similar to the ROLARR and Kim trials in both of our groups, median (IQR) 18 (13, 260) and 19 (14, 26) (learning, proficiency, respectively) vs 23.2 and 18 [17, 18].

No significant differences were noted between the study groups regarding post-operative complications or 30-day outcomes (Table 5). The rate of AKI in the learning group was higher (13.9% vs 3.9%) and approached significance (*p* = 0.055). This difference could be related to changes in the pre-operative optimization and the entire perioperative pathway that has been established over the study time frame. The cardiac complications recorded were all for atrial fibrillation except one hypertensive urgency in the learning group. The 30-day re-operation rate was 13 0.7% in the proficiency phase. Review of all operative notes revealed that all 7 cases were exams under anesthesia with drain placement if indicated for concern for anastomotic leaks. None in this group required any major abdominal re-operation.

Our leak rate was 5.1% and 7.8% in the learning and proficiency groups, respectively, compared to 14.8% (ROLARR) and 12.1% (Kim). SSI rates were also lower, 1.3% and 0%, compared with 8.9% (ROLARR) and 1.5% (Kim). Average length of stay (LOS) for the RCTs were 8.0 (ROLARR) and 10.3 days (Kim), vs median (IQR) 4 (3, 6) and 4 (3, 5) days in this study. Nearly all patients in the Kim study had a DLI (98.5%), whereas 60.2% of the patients in the ROLARR trial underwent DLI [17, 18].

A small study by a single surgeon in a community hospital showed similar outcomes after minimally invasive rectal resection compared with larger institutions, pointing out that perhaps the support and availability of a multidisciplinary team matters more than the hospital size/classification itself [19]. We believe this is also reflected in our data with outcomes at a community teaching center that are very consistent with previously published large series. In addition, the learning curve, specifically the learning phase, did not influence the outcomes negatively, showing the safety of performing rectal cancer care in the community and teaching setting. We must note we do have an established multi-disciplinary team of specialized providers in rectal cancer management and have set standards for pathologic and radiologic synoptic reporting, which have certainly helped in ensuring delivery of care along nationally established guidelines.

A review of nearly 24,000 patients who underwent rectal resection (open, laparoscopic, or robotic) was performed using the National Cancer Database to assess for predictors of robotic utilization for rectal cancer resection. They found that younger patients at academic institutions were significantly more likely to have a robotic rectal resection compared to open or laparoscopic [20]. We believe that based on our data this can be extended to community hospitals with well-trained robotic colorectal surgeons.

Limitations of this study include its retrospective nature and the evaluation of only a single surgeon’s outcomes. The latter was unavoidable since our institution only has one colorectal surgeon who routinely performs colorectal resections using the robotic platform. Also, there were many incomplete/non-standardized pathology reports in the learning phase prior to establishing the synoptic reporting, which can certainly influence the data reporting.

## Conclusions

This retrospective review of the learning curve for robotic rectal resections at a community teaching institution shows that throughout a learning curve, oncologic and post-operative outcomes are similar to those from previously reported large studies.
